# Proteomic insights into early pancreatic ductal adenocarcinoma biology and screening

**DOI:** 10.1007/s12672-025-03317-1

**Published:** 2025-08-11

**Authors:** Yue Huang, Chengzhi Sun, Xiangyuan Gao, Siqi Zhai, Guosheng Wang, Fan Zhang

**Affiliations:** 1 Department of Epidemiology and Biostatistics, School of Public Health, Shandong Second Medical University, Weifang, 261053 People’s Republic of China; 2 NHC Key Laboratory of Cell Transplantation, The First Affiliated Hospital of Harbin Medical University, Harbin, 150001 People’s Republic of China; 3 State Key Laboratory of Urban Water Resource and Environment, School of Chemistry and Chemical Engineering, Harbin Institute of Technology, Harbin, 150001 People’s Republic of China

**Keywords:** Early pancreatic ductal adenocarcinoma, Protein, Paired design, Risk model, Mendelian randomization

## Abstract

Pancreatic ductal adenocarcinoma (PDAC) presents significant diagnostic challenges at early stages due to the absence of specific symptoms and the rapid progression of the disease. Consequently, there is an urgent imperative to investigate the mechanisms underlying early detection and the biological processes that drive cancer progression. In response to this need, we conducted a paired design study employing differential protein analysis, Mendelian randomization (MR), and single-cell analysis to identify distinctive features associated with early-stage PDAC (E-PDAC). Our initial analysis in the RJ cohort identified 1,068 E-PDAC-related proteins from differential protein analysis. Subsequently, we employed a random forest approach to pinpoint 25 E-PDAC-specific proteins. These proteins informed the development of 13 machine learning models aimed at predicting E-PDAC risk, which demonstrated an area under the curve (AUC) of approximately 0.9 in the discovery cohort and approximately 0.8 in external validation. Furthermore, MR and single-cell analysis were utilized to explore causal relationships and the composition of the tumor microenvironment. Through MR, we identified STX7 as a risk factor (odds ratio = 1.26; confidence interval: 1.03–1.54; *p* = 0.02), with LUM exhibiting a dual role (pro-tumorigenic in proteomic analysis but anti-tumorigenic in MR analysis). Single-cell analysis revealed that LUM primarily aids in the generation of fibroblasts and T/B cells, serving as pro-tumorigenic and antitumorigenic agents, respectively. Our research offers valuable insights into protein biomarkers, cell types, and communication in E-PDAC, suggesting potential targets to improve screening efficiency.

**Supplementary Information:**

The online version contains supplementary material available at 10.1007/s12672-025-03317-1.

## Introduction

Pancreatic ductal adenocarcinoma (PDAC)is an aggressive disease with high surgical mortality and a 5-year survival rate often below 1%, making it the cancer with the worst prognosis [1, 2]. Studies have linked its development to factors like smoking, diabetes [3, 4], obesity [5, 6] and chronic pancreatitis [7]. About 10% of cases have a genetic basis, involving mutations in genes such as CDKN2A, STK11, and PRSS1 [6, 8]. Other contributing mechanisms include chromosomal changes, epigenetic shifts, and transcriptional alterations [9]. By 2030, pancreatic cancer is expected to become the second leading cause of cancer death in the United States [10]. PDAC is highly lethal primarily because it’s often detected late, after metastasis, due to its asymptomatic or vague symptom presentation. The pancreas’s location hinders routine screenings, and the lack of early-stage diagnostic biomarkers complicates the detection of localized tumor.

Pancreatic invasive neoplasia can take years to develop, offering a window for early detection, especially in high-risk groups [11, 12]. Biomarkers might help identify high-grade precursors or early-stage pancreatic cancer. Some labs have screened accessible biological samples, finding TP53/SMAD4 mutations in pancreatic juice as PDAC risk factors [13]. Other biomarkers in PDAC patients’ blood include CA19-9, HbA1C, tumor markers [14–16], circulating tumor cells [17], exosomes, circulating-tumor DNA, or cell-free DNA [18]. However, low levels of peripheral DNA limit sensitivity in early-stage PDAC detection. CA19-9 is the most used diagnostic biomarker, despite its limited sensitivity. CA19-9 remains the most commonly used diagnostic biomarker for PDAC, despite its sub-optimal sensitivity. It is important to note that any biomarker initiative for PDAC will inevitably result in false positives within the average-risk population, necessitating exceptionally high specificity and sensitivity. Investigating the molecular basis of PDAC is crucial. Proteins, key to biological functions and traits, provide valuable insights when measured directly. Researchers have effectively used proteome-focused cross-omics analyses to reveal tumor composition differences [19, 20]. The PDAC tumor microenvironment features abundant fibroblasts, a dense extracellular matrix, poor vascularization, and a varied, mainly immunosuppressive cell population. Understanding changes and cell interactions in E-PDAC stages is vital.

**Fig. 1:**
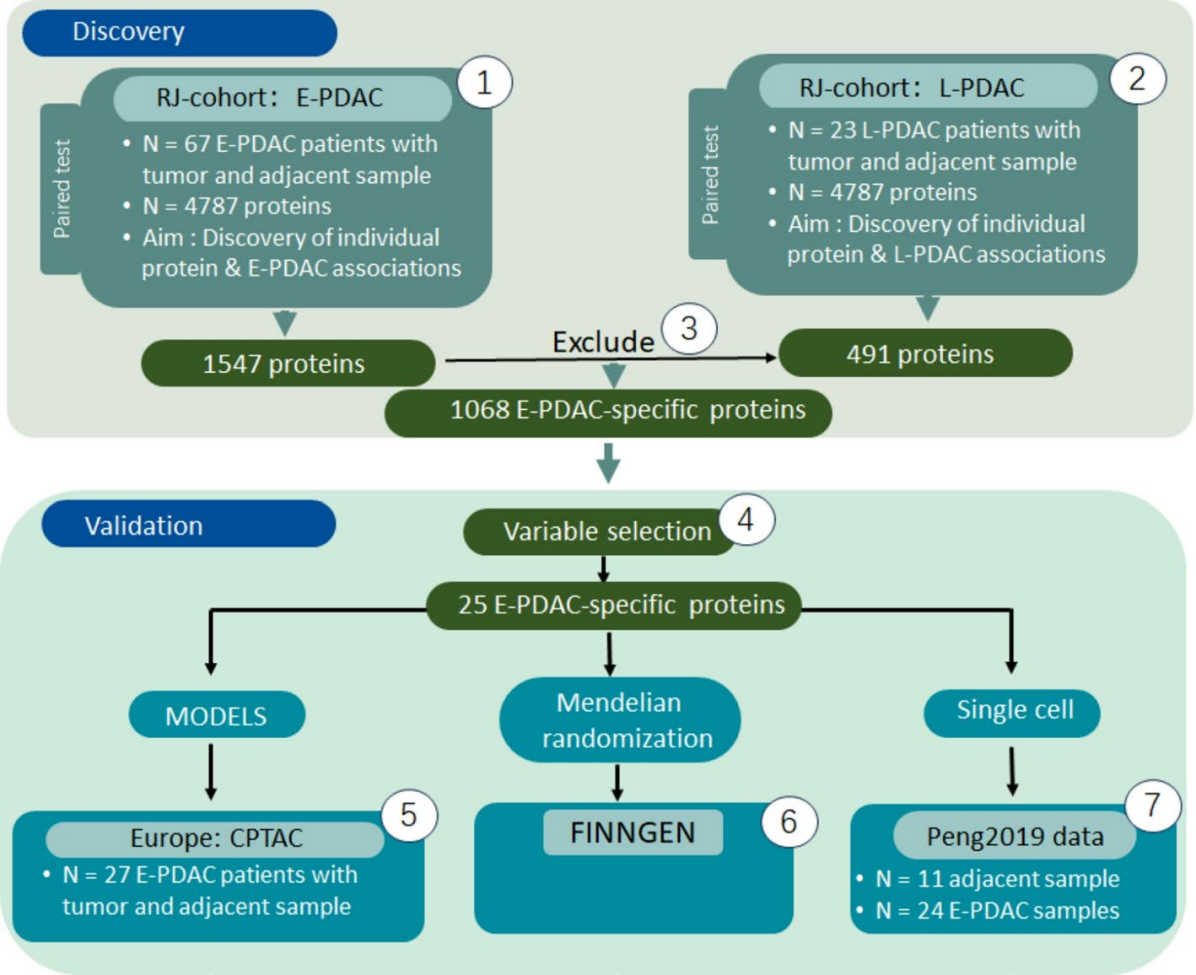
Research workflow diagram

Our investigation of E-PDAC was organized around several principal objectives. Initially, we identified and validated 25 E-PDAC-specific proteins which were potential biomarkers and therapeutic targets, utilizing a paired design approach. Subsequently, we developed a predictive model for E-PDAC screening, which exhibited robust performance metrics. Furthermore, through Mendelian randomization analyses, we identified the proteins STX7 and LUM as potential factors for PDAC. Additionally, we conducted a comparative analysis of the PDAC tumor microenvironment across tumor-adjacent normal tissue, as well as early and late stages of the disease, leading to the identification of an E-PDAC-specific cell type. The workflow of the entire study is illustrated in Fig. 1.

## Materials and methods

### Data source

Data collection and sources data are drawn from a range of sources, and multi-omics data were used in this research, including genome, transcriptome and proteome Table 1. For proteome data, the RJ-cohort data [21] and CPTAC data [22] were seen as the discovery set and validation set separately. RJ-cohort was obtained from Shanghai Jiao Tong University Ruijin Hospital, which has made 281 samples * 4784 proteins data available publicly. In the CPTAC data, a total of 149 samples were collected in our study, which including 44 tumor adjacent samples and 105 PDAC tumor samples. The PDAC GWAS summary data was obtained from FinnGen research project [23], which aimed to identify genotype-phenotype correlations in the Finnish population. For the proteomic GWAS summary data, we integrated seven large-scaled proteomic studies (Pietzner et al., 4775 proteins [24]; Ferkingstad et al., 4719 proteins [25]; Sun_1 et al., 2995 proteins [26]; Sun_2 et al., 1463 proteins [27]; Suhre et al., 1124 proteins [28]; Folkersen et al., 90 proteins [29]; Yao et al., 71 proteins [30]), and extracted summary statistics of genetic associations with plasma proteins. The PDAC single cell data was from Peng’s research [31], which collected the PDAC tumor samples and non-PDAC tumor samples from Peking Union Medical College Hospital.

**Table 1:** The data source

Data source	Data type	Sample size
RJ-cohort data	PDAC Protein data	281
CPTAC data	PDAC Protein data	149
FinnGen data	PDAC GWAS data	347,110
Pietzner et al.	Proteomic GWAS data	4775
Ferkingstad et al.	Proteomic GWAS data	4719
Sun_1 et al.	Proteomic GWAS data	2995
Sun_2 et al.	Proteomic GWAS data	1463
Suhre et al.	Proteomic GWAS data	1124
Folkersen et al.	Proteomic GWAS data	90
Yao et al.	Proteomic GWAS data	71
Peng et al.	PDAC single cell data	35

### Differential protein analysis of the paired design

In this study, the RJ cohort was discovery set, and CPTAC was validation set. Firstly, we named E-PDAC or L-PDAC based on their clinical stage. Specifically, stages I/IIa (or I/II if stage II was not further subdivided) were classified as E-PDAC, while the remaining stages were designated as L-PDAC. Samples from adjacent non-tumorous tissue were labeled as Normal stage. Secondly, the paired design was conducted in discovery and validation sets. From the RJ cohort, comprising 281 samples, 67 E-PDAC & Normal tissue pairs, as well as 23 L-PDAC & Normal tissue pairs, were identified. Additionally, 27 E-PDAC & Normal tissue pairs from the CPTAC dataset were utilized to validate the findings from the RJ cohort. Thirdly, differential protein screening analysis was conducted on both the E-PDAC & Normal pairs and the L-PDAC & Normal pairs, and the E-PDAC-related proteins and L-PDAC-related proteins were obtained. The E-PDAC-specific proteins are E-PDAC-related proteins excluding L-PDAC-related proteins. A paired Wilcoxon rank-sum test was employed for statistical analysis, and all P-values were adjusted using the Bonferroni correction method.

### E-PDAC-specific protein identification and predictive model construction

To refine the E-PDAC-specific proteins obtained previously, a random forest algorithm was employed. Proteins exhibiting an importance value greater than 0.5 in the random forest analysis were designated as final E-PDAC-specific proteins. Utilizing these key proteins, 13 distinct machine learning methods were employed to develop the E-PDAC predictive model based on 10-fold cross-validation. These methods included ranger, nnet, kknn, naïve Bayes, logistic regression, SVM, C5.0, XGBoost, logistic, LDA, GBM, LightGBM, and random forest. During the 10-fold cross-validation, the data is divided into 10 subsets, or “folds.” The model is trained on 9 of these folds and tested on the remaining fold. This process is repeated 10 times, with each fold serving as the test set once. The results are then averaged to provide an overall performance metric. The 10-fold cross-validation could balance between bias and variance in the model evaluation. The primary evaluation metrics comprised the area under the curve (AUC), accuracy (ACC), sensitivity, and specificity. The predictive models were trained using E-PDAC and normal paired data from the RJ cohort, while the CPTAC dataset served as external validation to assess model performance. The “mlr” R package was utilized for this analysis.

### Mendelian randomization

The MR analysis was performed to explore the relationship between proteomic data and PDAC based on the genome perspective. Out of seven large proteomic studies, only five plasma protein data of discovery results were found. The proteomic datasets were selected for inclusion in the analysis based on the following criteria [32]: (I) Genome-wide significance: Only pQTLs that reached the genome-wide significance threshold (*P* < 5 × 10⁻⁸) were included to ensure the statistical robustness of the identified associations; (II) Exclusion of MHC regions: pQTLs located within the major histocompatibility complex (MHC, located in the 26–34 Mb region of chromosome 6) were excluded to reduce the potential interference of this highly polymorphic region on the results; (III) Linkage disequilibrium filtering: pQTLs with significant linkage disequilibrium (LD, r² < 0.001) were further screened to ensure the independence of the selected instrumental variables; (IV) F-statistic threshold: To ensure the strength of instrumental variables, we only keep the corresponding pQTL for each protein with an F-statistic greater than 10, thereby minimizing the bias introduced by weak instrumental variables. The PDAC GWAS summary was from the FinnGen database, comprising 1,992 pancreatic cancer cases and 345,118 control samples.

### Single cell analysis of PDAC

Single-cell RNA sequencing data from PDAC, as reported in Peng et al. [33], was utilized to investigate the influence of E-PDAC-associated markers on the risk of E-PDAC. The cell types, as annotated by the original authors, were employed without further clustering. Initially, we analyzed the variations in cell type distribution across Normal, Early, and Late stages. Subsequently, we sought to identify stage-specific cell types and examined their relationship with E-PDAC. Finally, CellChat analysis was conducted to explore intercellular communication among various cell types, with a particular focus on stage-specific cells.

## Results

### Proteome-wide differential analysis identification for E-PDAC

**Fig. 2:**
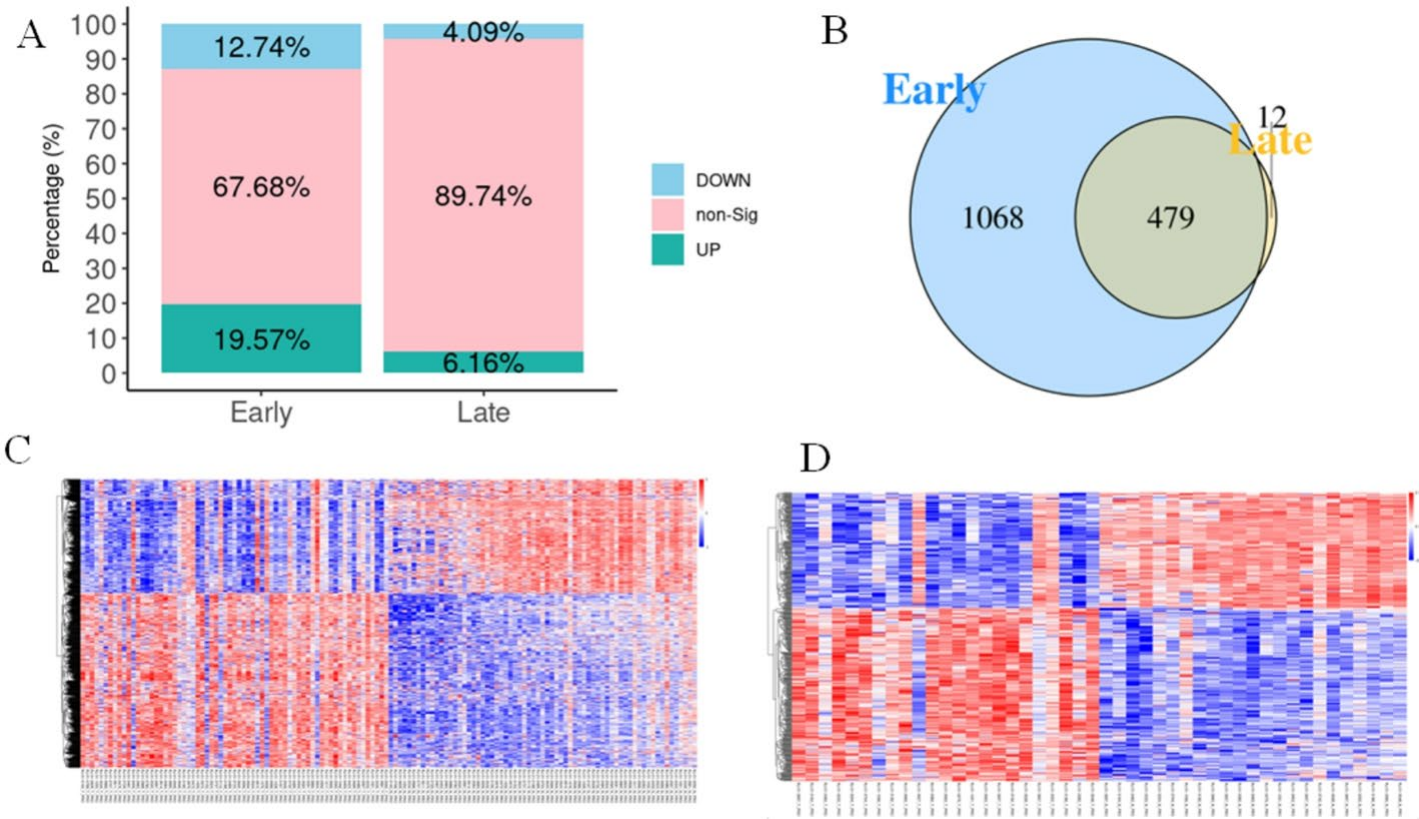
Differential proteins between different stages in PDAC.** A** The role of differential proteins in Early and Late stage; **B** Wayne diagram of differential proteins in two stages; The heatmap of differential proteins in Early (**C**) and Late (**D**) stage. Up-regulated proteins were shown in red and down-regulated proteins were shown in blue

In this study, we sought to identify differential proteins associated with E-PDAC using data from the RJ cohort. Based on clinical information, patients with stage I and II PDAC were classified as E-PDAC, while those with more advanced stages were categorized as L-PDAC. To identify the most influential proteins in E-PDAC based on paired design, we selected PDAC patients who provided both tumor-adjacent normal and tumor samples. This selection resulted in 67 paired E-PDAC&N samples, and 23 paired L-PDAC&N samples. Paired analyses were conducted using the Wilcoxon signed-rank test. In the E-PDAC paired analysis, the proportions of differentially up-regulated, down-regulated, and non-differential proteins were 19.57%, 12.74%, and 67.68%, respectively (Fig. 2A). In contrast, the L-PDAC paired analysis revealed proportions of 6.16%, 4.09%, and 89.74% for up-regulated, down-regulated, and non-differential proteins, respectively (Fig. 2A). Overall, 1547 proteins were identified as differential variables in the E-PDAC analysis, while 491 proteins were identified in the L-PDAC analysis (Fig. 2B). The intersection of these analyses indicated that 479 proteins were common to both. The number of proteins related to E-PDAC and L-PDAC were 1,068 and 12, respectively. Additionally, we presented heatmaps illustrating the differential proteins for both paired designs (Fig. 2C, D).

### 25 E-PDAC-specific protein identification and 13 predictive models construction

**Fig. 3:**
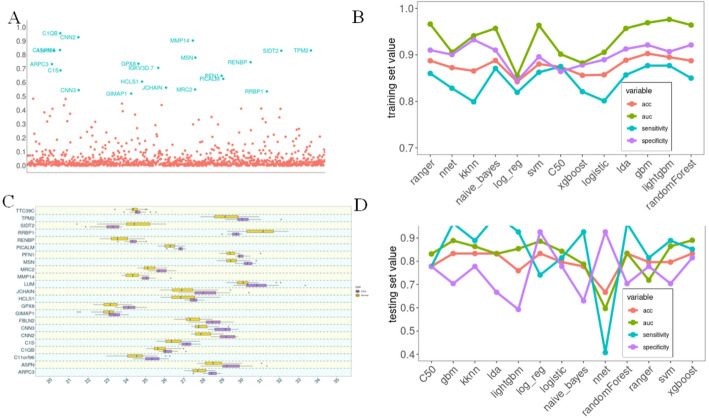
E-PDAC-related signature in discovery and validation set.** A** Point plot of E-PDAC-related signature, which was in red. The 13 models’ performance in discovery (**B**) and validation (**D**) set.** C** The boxplot of E-PDAC-related signature in validation set

In this part, we aimed to narrow down the E-PDAC related proteins using random forest to find out the most accurate E-PDAC-specific protein. According to the feature importance scores given in the random forest analysis, the proteins with importance value greater than 0.5 were named as E-PDAC-specific protein. In this way, we obtained 25 proteins which were strongly link with E-PDAC, including TPM2, SIDT2, RRBP1, RENBP, PFN1, PICALM, MMP14, MSN, MRC2, GPX8, IGKV3D.7, HCLS1, JCHAIN, GIMAP1, TTC39C, TPM2, LUM, FBLN2, CNN3, C1S, ARPC3, CNN2, C1QB, C11orf96 and ASPN (Fig. 3A). Based on these 25 E-PDAC-specific proteins, the 13 machine learning algorithms were used to construct models for E-PDAC prediction. We trained and validated our models using 10 folds cross-validation based on the E-PDAC data of RJ-cohort. As Fig. 3B showed, AUC, ACC, sensitivity and specificity were almost greater than 0.8. The AUC performance of ranger, naïve Bayes, SVM, LDA, GBM, lightGBM and random forest was relatively better, with more than 0.9. All seven of the above machine learning methods showed a high accuracy of more than 0.85. In addition, the specificity was intermediate between the two. In contrast, although the sensitivity of these machine learning methods was relatively low, it still exhibited greater than 0.8.

### External validation of 25 E-PDAC-specific proteins and 13 predictive models

This part served as an in-depth verification of findings based on the external validation data. In our study, the E-PDAC protein data of CPTAC dataset were treated as the external validation set. Similar with RJ-cohort, we named stage I and stage II as E-PDAC to analyze more granularly, and we collected 27 patients for paired E-PDAC analysis. Unfortunately, two E-PDAC-specific proteins were missing from the CPTAC dataset, and only 23 E-PDAC-specific proteins were found. In order to maintain the consistency of variables, we rebuilt prediction models using these 23 proteins based on RJ-cohort. Then these models were validated on the CPTAC dataset. The performance of validation has somewhat larger fluctuations than the discovery set. As Fig. 3C showed, the AUC values were greater than 0.8 in the most of models, such as C50, GBM, KKNN, LDA, lightGBM, Random Forest, SVM, and XGboost. Similar trends were observed in ACC performance. Compared to the AUC and ACC, the sensitivity and specificity showed fewer stable performances, which might be related to the smaller sample size. Beyond that, we carried out the paired sum-rank test on 23 E-PDAC related proteins in CPTAC data. As our expected, all the proteins’ difference between the E-PDAC and Normal was statistically significant (Fig. 3D). These results further verified the aforementioned findings. As anticipated, the variations among the 23 E-PDAC-specific proteins were statistically significant (Fig. 3D), thereby providing additional validation for the aforementioned findings.

### Proteome-wide MR analysis verified two proteins

**Fig. 4:**
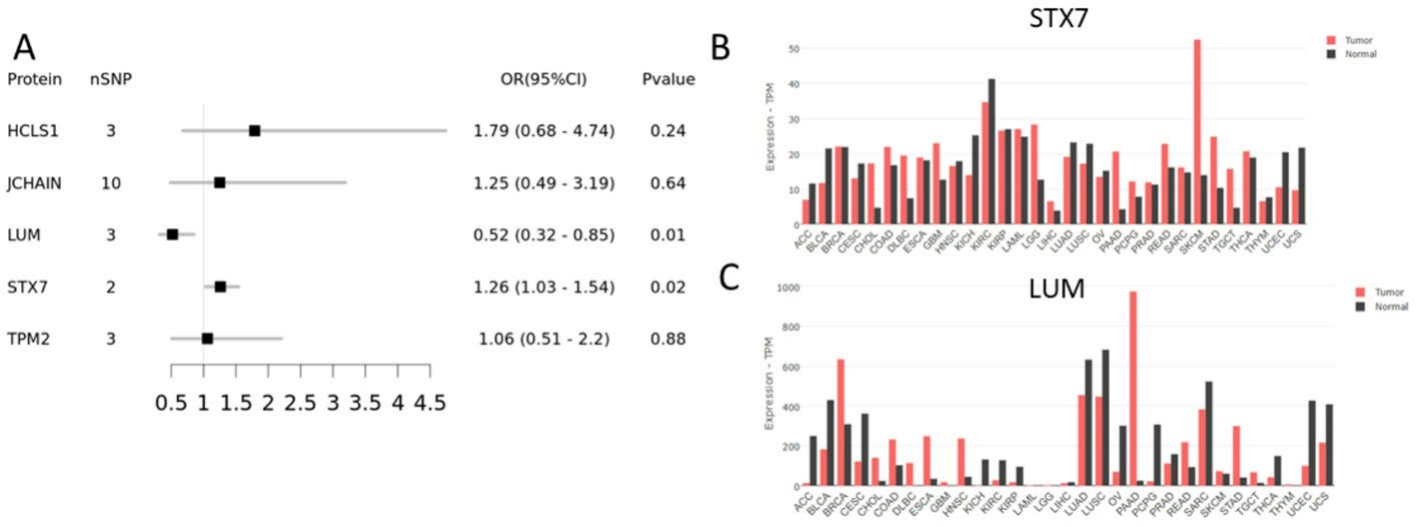
Mendelian randomization analysis result of E-PDAC-related signature. **A** The forest plot of E-PDAC-related signature. The gene expression of STX7 (**B**) and LUM (**C**) in pan-cancer

To further verify the observed findings, we performed MR analysis for E-PDAC-specific proteins with full summary-level data. 5 protein GWAS summary data were found from seven large-scaled proteomic studies. According to the Wald ratio and IVW results, 2 proteins (STX7 and LUM) were significantly related to PDAC risk (Fig. 4A). Genetically predicted higher levels of STX7 were associated with an increased risk of PDAC (OR = 1.26; 95%CI:1.03–1.54; *p* = 0.02), while the LUM proteins were negatively associated with PDAC risk (OR = 0.52; 95%CI:0.32–0.85; *p* = 0.01). No causal or correlations relationship was found between the other three proteins (HCLS1, JCHAIN and TPM2) and PDAC. To further confirm the conclusion of genetic MR analysis, we compared the expression of STX7 and LUM in paired human cancer specimens and adjacent non-cancerous tissues based on TCGA by GEPIA 2.0 (Fig. 4B, C). As expected, we observed the expression of STX7 in PDAC is higher than cancer adjacent, which was consistent with our previous findings and suggested STX7 is the risk factor of PDAC (Fig. 4B). Surprisingly, we observed the opposite LUM effect compared to the previous one. From TCGA transcriptome differential analysis, the expression of LUM in the PDAC is much higher than tumor adjacent non-cancerous tissues. This difference pattern was most apparent in the pan-cancer analysis (Fig. 4C). LUM, also known as Lumican, is an extracellular matrix protein, and the effects of LUM on PDAC warrant further investigation.

### ScRNA-seq analysis uncovered the cell type enrichment of E-PDAC-specific proteins

**Fig. 5:**
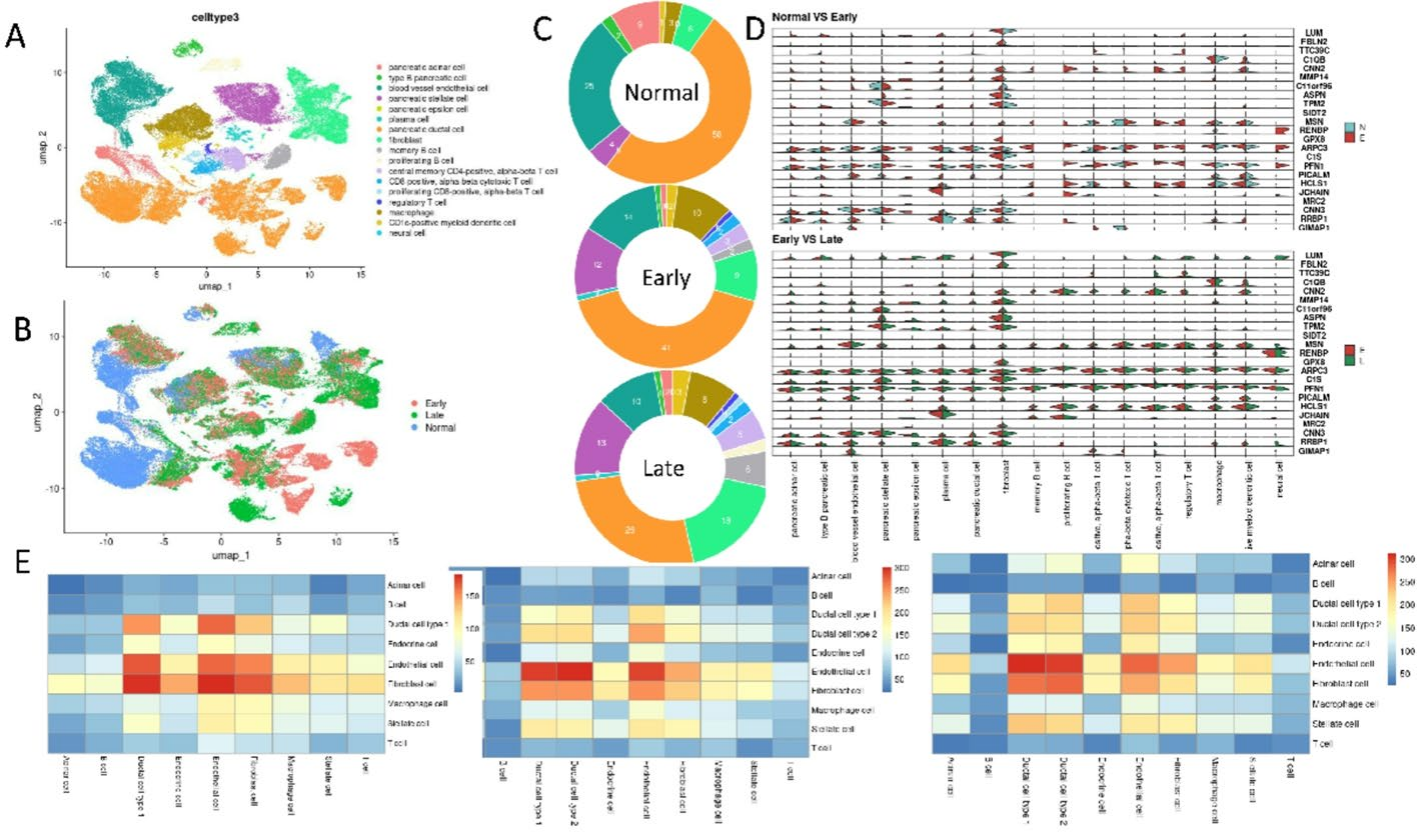
Single-cell analysis of PDAC. The cell type (**A**) and stage (**B**) distribution of PDAC. **C** The cell type proportion in Normal, Early and Late PDAC.** D** The violin plot of E-PDAC signature in Early and Late PDAC. (E) The cell communication of Normal, Early and Late PDAC

To delve deeper into the mechanism of E-PDAC progression, we implemented scRNA-seq analysis to illustrate the cell type distribution based on E-PDAC-specific proteins. The Peng’s data provided the rich information in cell annotation, and we no longer re-annotated each single cell. Different from RJ-cohort described earlier, the stage II was divided into IIA and IIB. Therefore, we named stage I and stage IIA as E-PDAC, and remaining was seen as L-PDAC. Finally, a total of 11 normal samples, E-PDAC samples and L-PDAC samples were collected, which covered 57,423 cells. These cells partially derived from the pancreatic cell, partially from blood vessel, partially from fibroblast, and partially from immune cells (Fig. 5A). Here, these cells were presented from PDAC stage perspective (Fig. 5B), including Normal, E-PDAC and L-PDAC. Considering Fig. 5A, B, we found that some cell types presented throughout three stages, such as pancreatic ductal cell. Interestingly, some cell was only seen in the early or late stage, such as subset of the pancreatic ductal cell and fibroblast cell. As the disease progresses, changes occur in endothelial and pancreatic cells, suggesting heterogeneity within these cell types, which can be further subdivided. If true, we would expect stage-specific cell subgroups. In normal stages, cells are mainly in the top-left area, while early-stage cells are in the bottom-right. Figure 5C shows cell type proportions at different stages. With disease progression, the proportions of pancreatic stellate, fibroblast, and immune cells increase, while blood vessel endothelial, pancreatic acinar, and ductal cells decrease.

To associate E-PDAC signatures with cell types, we plotted violin graphs for Normal, Early, and Late stages (Fig. 5D). Pancreatic epsilon cells appeared in the early stage but were absent in the normal stage (Table S1), similar to memory B cells, proliferating B cells, CD8 T cells, regulatory T cells, and neural cells. Genes LUM, MMP14, ARPC3, PICALM, and CNN3 were enriched in pancreatic epsilon cells, while CNN2, MSN, ARPC3, C1S, PFN1, and HCLS1 were enriched in T and B cells. These changes were not observed in the L-PDAC phase. The LUM gene was mainly found in fibroblasts during the normal stage but became enriched in immune cells, including T and B cells, as the disease progressed. We also examined cell communication differences across stages (Fig. 5E), noting increased cell-cell communication with PDAC progression. In the normal stage, ductal, endocrine, and endothelial cells communicated more frequently, a pattern that persisted throughout the PDAC stages. The interaction between acinar cells and other cell types was observed to intensify, with a similar trend noted in T and B lymphocytes. This suggests a progressive strengthening of intercellular communication, whereby the cells increasingly exert influence on one another.

## Discussion

In this study, we performed a comprehensive multi-omics analysis, including proteomics, genomics, and transcriptomics, to investigate E-PDAC. We identified 25 E-PDAC-specific proteins by comparing tumor and adjacent normal tissues. From this, we developed 13 predictive models and validated them with an external dataset. MR analysis, using genome-wide association study data, linked two proteins, LUM and STX7, to PDAC. ScRNA analysis showed changes in cell type proportions as PDAC progressed, with increased immune cells. LUM was expressed in fibroblasts in normal stages but found in immune cells in both E-PDAC and L-PDAC.

The tumor microenvironment (TME) of PDAC is complex and significantly influences disease progression and treatment resistance. LUM, a proteoglycan that regulates collagen fibril assembly, is found in the PDAC extracellular matrix and is associated with improved patient outcomes after therapy and surgery. Activated pancreatic stellate cells are primary producers of LUM, but its secretion is inhibited by TGF-β through SMAD binding elements in the LUM gene promoter, indicating LUM’s role in ECM modulation and stromal behavior [34]. Additionally, LUM affects immune cell infiltration, potentially contributing to PDAC’s immunosuppressive environment and impacting immunotherapy effectiveness [35]. Its interactions with cancer-associated fibroblasts (CAFs), which drive the desmoplastic reaction in PDAC, may also influence tumor progression, suggesting that targeting specific CAF subtypes alongside LUM could be a promising therapeutic strategy. LUM’s interaction with pathways like PI3K-AKT may uncover mechanisms of tumor progression and therapy resistance, emphasizing the ECM’s role in cancer [36]. While LUM shows promise as a PDAC biomarker due to its involvement in the tumor microenvironment and key signaling pathways, further research is needed to fully understand its diagnostic and prognostic value. Combining LUM with other biomarkers like CA19-9 and microRNAs could enhance early detection and treatment strategies for PDAC.

The LUM’s role in proteomic and MR analysis differed due to two main reasons. First, protein post-translational modifications, like phosphorylation, can alter protein interactions, potentially impairing tumor suppressor functions and even promoting cancer, as seen with the p53 protein [37]. Second, the tumor microenvironment can influence gene roles, with proteins from genes initially seen as tumor suppressors potentially promoting tumor growth and migration in breast cancer due to factors secreted by tumor-associated macrophages [38]. LUM is a common ECM component that organizes the collagen matrix and influences cell proliferation signals in cancer. In cancer biology, LUM expression in the tumor microenvironment can have either pro-tumorigenic or anti-tumorigenic effects. Its role varies with the stroma-rich tumor microenvironment, involving the activation of FAK, MAPK, and MMP-9. High levels of LUM in cancer tissue may lead to chemoresistance, increasing malignancy risk and potentially promoting tumor growth [39].

Our study found distinct cell type changes during PDAC research. In the normal phase, pancreatic ductal and blood vessel endothelial cells were prevalent. There was a marked increase in fibroblast and immune cells in E-PDAC, which continued into L-PDAC. However, the rise in pancreatic stellate cells seen in E-PDAC did not persist in L-PDAC. As the disease advances, E-PDAC-related signatures within cell types change, altering the tumor microenvironment. Research indicates that cells in this environment can work together to promote tumor development [40, 41]. With advancements in cancer treatment, targeting intercellular communication is crucial, not just focusing on cell types or counts. Enhanced communication between acinar, endothelial, and ductal cells has been observed. Adult pancreatic acinar cells can transform into progenitor-like cells with ductal traits, aiding in pancreatic regeneration after injury [42]. This phenomenon also offers theoretical support for the targeted treatment of PDAC.

Our study identified E-PDAC-specific proteins (LUM and STX7) via multi-omics analysis, offering new insights into its etiology and early detection. Further research is needed to evaluate these proteins’ utility and validate our findings.

### Limitations

A key strength of this study is its paired design in discovering E-PDAC-related signatures, reducing confounding factors. However, improvements are needed: increasing the PDAC proteomic sample size for broader validation, dynamically measuring plasma protein levels with trajectory analysis, and addressing potential group stratification bias from merging GWAS data. Ultimately, the study’s results will aid in developing rapid detection kits.

## Conclusions

Our analysis identified 25 E-PDAC-specific proteins using paired differential proteins analysis and random forest approach. Based on these proteins, we developed 13 machine learning models to predict E-PDAC risk, achieving an AUC of ~ 0.9 in the discovery cohort and ~ 0.8 in external validation. Using MR and single-cell analysis, we found STX7 as a risk factor (OR = 1.26), and LUM as dual role (pro-tumorigenic in proteomic analysis but anti-tumorigenic in MR analysis). Single-cell analysis further clarified that LUM primarily facilitates the development of fibroblasts and T/B cells, which demonstrate pro-tumorigenic and antitumorigenic effects, respectively. Collectively, our extensive proteomic, Mendelian randomization, and single-cell analyses offer significant insights into the molecular mechanisms underlying E-PDAC.

## Supplementary Information

Additional file 1.

## Data Availability

All data generated or analyzed during this study are included in published article and its supplementary information files.
