# Supplementary material for: Proteomic insights into early pancreatic ductal adenocarcinoma biology and screening
Source: Discov Oncol. 2025 Aug 11;16:1531. doi: 10.1007/s12672-025-03317-1 (PMC12339791; doi:10.1007/s12672-025-03317-1)
Supplement: Supplementary file 1 — Additional file 1. [file 12672_2025_3317_MOESM1_ESM.docx]

Table S1 The celltype distribution among different stage of PDAC

|  | Normal |  | Early |  | Late |  |
| --- | --- | --- | --- | --- | --- | --- |
| celltype | Count | Percent(%) | Count | Percent(%) | Count | Percent(%) |
| pancreatic acinar cell | 1365 | 9 | 142 | 1 | 541 | 2 |
| type B pancreatic cell | 270 | 2 | 183 | 1 | 128 | 1 |
| blood vessel endothelial cell | 3922 | 25 | 2688 | 14 | 2417 | 10 |
| pancreatic stellate cell | 583 | 4 | 2313 | 12 | 2966 | 13 |
| pancreatic epsilon cell | 0 | 0 | 3 | 0 | 37 | 0 |
| plasma cell | 29 | 0 | 243 | 1 | 277 | 1 |
| pancreatic ductal cell | 7770 | 50 | 7653 | 41 | 6064 | 26 |
| fibroblast | 940 | 6 | 1727 | 9 | 4259 | 18 |
| memory B cell | 1 | 0 | 330 | 2 | 1466 | 6 |
| proliferating B cell | 0 | 0 | 90 | 0 | 540 | 2 |
| central memory CD4-positive, alpha-beta T cell | 3 | 0 | 579 | 3 | 1110 | 5 |
| CD8-positive, alpha-beta cytotoxic T cell | 13 | 0 | 358 | 2 | 431 | 2 |
| proliferating CD8-positive, alpha-beta T cell | 1 | 0 | 91 | 0 | 156 | 1 |
| regulatory T cell | 0 | 0 | 96 | 1 | 116 | 1 |
| macrophage | 476 | 3 | 1958 | 10 | 1903 | 8 |
| CD1c-positive myeloid dendritic cell | 86 | 1 | 414 | 2 | 637 | 3 |
| neural cell | 0 | 0 | 15 | 0 | 33 | 0 |
